# Drug–microbiome interactions: What we know and why predictive translation remains elusive

**DOI:** 10.1080/29933935.2026.2649166

**Published:** 2026-04-01

**Authors:** Fatima C. Pereira, Sahar El Aidy

**Affiliations:** a School of Biological Sciences, Faculty of Environmental and Life Sciences, University of Southampton, Southampton, United Kingdom; b Department of Microbiome Engineering, Swammerdam Institute for Life Sciences, University of Amsterdam, Amsterdam, The Netherlands; c The Holomicrobiome Institute, Amsterdam, The Netherlands

**Keywords:** Drug–microbiome interactions, functional readouts, stratification, ecological dynamics, pharmacomicrobiomics

## Abstract

Medication-induced alterations of the gut microbiome influence drug efficacy, toxicity, and long-term outcomes. Despite extensive evidence for drug-microbe interactions, predictive translation into clinical practice remains limited. Generalization from shifts in taxonomic profiles, mechanistic studies or isolated enzymatic assays is challenging because microbial activity is highly context-dependent. Drug-microbiome interactions are shaped by host factors including pH, transit time, nutrient and cofactor availability, and spatial organization along the gastrointestinal tract. Here, we argue that predictive translation requires measuring functional outputs, site-specific activity, ecological interactions, and host-contextual modulation, rather than static microbial properties.

## Introduction

**Figure 1.**
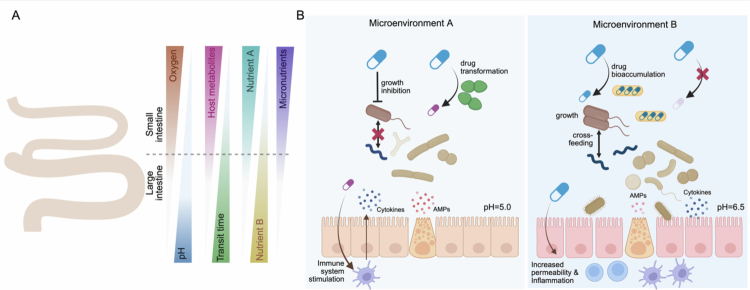
Ecological dynamics of drug-microbiome interactions. **A.** Intestinal gradients of oxygen, pH, transit time, host metabolites, and nutrients (*e.g.,* nutrients A and B, micronutrients) create distinct microenvironments along the human gut. **B.** These microenvironments shape drug-microbe interactions. In microenvironment A, low pH favors the growth of microbes capable of drug biotransformation, generating drug variants that can trigger inflammation and cytokine release from the host, thereby influencing microbiome composition. High local concentrations of drugs with antimicrobial properties can directly inhibit microbial growth or disrupt community behaviors such as cross-feeding, further modifying the microbiome. In microenvironment B, the presence of a drug bioaccumulating species can lower local drug concentrations, permit growth of otherwise inhibited microbes and enable alternative community states through renewed cross-feeding. Additionally, some drugs increase intestinal permeability and modulate immune responses, further altering community composition. These microenvironments can coexist or alternate over time within the gut, shaping microbial community composition as well as the spatial and temporal patterns of drug availability to the host. Created in Biorender.

Medications, particularly those taken orally, typically come into direct contact with the gut microbiome, producing functional changes that can influence treatment outcomes.^1‐4^ Drugs such as antidiabetics, proton pump inhibitors, psychotropics (including antidepressants and antipsychotics), GLP-1 receptor agonizts, or statins frequently alter gut microbial composition,^1^^,^^2^^,^^4^^,^^5^ and can, in some cases, increase diversity and improve clinical outcomes, particularly in combination therapies.^4^ While these interactions are increasingly recognized, their clinical predictability remains limited. In practice, some patients exhibit clear microbiome-mediated drug responses, yet attempts to generalize findings across populations often fail. This reflects not a lack of evidence, but rather the mismatch between how interactions are measured and the factors that determine clinical outcomes. Drug-microbe interactions are usually studied in isolation; *in vitro*, in monocultures, or in well-mixed, reduced-complexity communities, ignoring host-context dependence, spatial heterogeneity, and ecological dynamics that govern microbial function *in vivo*. Mechanistic studies demonstrate the diverse nature of drug–microbe interactions and have yielded important insights into the molecular basis of microbial drug metabolism. However, translating these discoveries into predictive clinical markers remains challenging because mechanistic findings obtained in simplified systems do not always generalize to the complex gut ecosystem. Three systematic limitations contribute to this gap: (i) *Mechanistic reductionism*: while studies focusing on isolated enzymes or single-taxon have identified key pathways of drug transformation, their effects can be substantially modified by microbial community interactions *in vivo*; (ii) *Neglect of spatial structure*: overlooking differences along the gastrointestinal tract where microbial density, function, and drug exposure vary dramatically; (iii) *Ignoring host and ecological context*: pH, nutrient fluxes, cofactors, mucus, transit times, and interspecies interactions dynamically shape microbial enzymatic activity (Figure 1). Addressing these limitations is critical to move from reporting the existence of drug–microbe interactions to enabling predictive, patient-specific therapeutic guidance.

## Results

### Mechanistic insights: direct and indirect interactions

Human-targeted drugs shape gut microbiome composition through direct and indirect mechanisms. Large-scale *in vitro* screens of 835 drugs demonstrated that ~24% directly inhibit microbial growth.^6^ Direct mechanisms include membrane disruption, modulation of efflux pumps, interference with nutrient acquisition, or inhibition of ribosome biogenesis.^7^ Drug–microbiome interactions are reciprocal: gut microbes can also metabolize or chemically modify pharmaceutical drugs, altering efficacy or generating toxic metabolites.^3^^,^^8^^,^^9^ Microbes can also bioaccumulate pharmaceuticals, modulating local exposure in a strain- and compound-specific manner.^10‐12^ However, outcomes of drug exposure on gut microbes depend strongly on microbial community context: cross-protection and cross-sensitization emerge when one taxon shields or sensitizes others to drug exposure (Figure 1B).^13^ Consequently, isolated mechanistic assays often fail to predict *in vivo* outcomes.

Indirect mechanisms further complicate predictions: drugs can alter luminal conditions, pH, mucus composition, epithelial permeability, and nutrient fluxes, thereby reshaping microbial colonization and metabolic activity (Figure 1B).^14^ Notably, pharmaceutical excipients can also modulate microbial composition and function,^15^ making it challenging to disentangle the effects of the active drug from those of added excipients and complicating the implementation of microbiome monitoring in clinical practice. These effects operate at the ecosystem level, emphasizing that function emerges from the interactions between the drug, the microbiome, and the host environment. Moreover, microbial enzymatic activity mediating drug metabolism is not an intrinsic property of a taxon alone, but depend on cofactor availability, transit dynamics and other host-context factors, including those described above.^16^

### Functional readouts outperform taxonomy

The gut microbiome plays a central role in determining host drug responses by biotransforming and bioaccumulating drugs, and by shifting its metabolism in response to drug exposure.^8,17‐20^ Human cohort studies demonstrate that taxonomy alone poorly predicts drug response. In oncology, baseline microbiome composition inconsistently correlates with immune checkpoint inhibitor response.^17^ By contrast, microbial metabolites and functional pathways show stronger, reproducible associations.^18^^,^^19^ Similarly, in rheumatoid arthritis, microbial metabolic potential distinguishes methotrexate responders from non-responders more reliably than species composition.^20^ These observations illustrate that functionally convergent outputs, rather than taxonomic profiles, are critical for stratifying patients, yet functional readouts remain underrepresented in clinical studies.

Metformin, a first-line therapy for type 2 diabetes, exemplifies a drug whose therapeutic effects are partly mediated by the gut microbiome. It modulates microbial metabolic pathways involved in short-chain fatty acid and bile acid metabolism, contributing to improved glucose homeostasis.^21^^,^^22^ Preclinical studies, particularly fecal microbiota transplantation in germ-free mice, have demonstrated that metformin-induced microbiome changes are sufficient to transfer drug benefits, providing direct evidence of a causal microbiome-mediated component of its action.^22^ In contrast, applying comparable experimental approaches to drugs targeting the central nervous system, such as antidepressants, is considerably more complex.^6^ Neuroactive drugs may influence the host through the gut-brain axis, potentially via microbiome‑dependent pathways,^6^ but the complexity of these pathways and the limited ability of models to capture human-relevant behavioral phenotypes make causal inference challenging.^23^

Evidence highlights the need to consider microbial metabolic outputs when translating mechanistic insights into outcomes that are predictive of clinical results. However, current models may be insufficient for establishing causality, particularly for certain drug classes.

### Spatial heterogeneity shapes drug fate

Along the gastrointestinal tract, gradients of pH, oxygen tension, nutrient availability, bile acids, host immune activity, and transit time generate a mosaic of distinct ecological niches (Figure 1). These spatially structured environments shape gut microbial biogeography and activity. Thus, drug–microbe interactions are spatially structured along the gastrointestinal tract. The small intestine, with a high surface area and permeability and low microbial density, is optimized for host-mediated absorption.^9^^,^^16^ Drugs absorbed proximally largely escape microbial transformation. In contrast, the distal ileum and colon harbor dense, functionally-specialized microbial communities enriched for reductive, deconjugating, and fermentative activities.^16^ This site-specific microbial enzymatic activity critically shapes drug fate: azoreductases activate sulfasalazine,^24^ *β*-glucuronidases promote irinotecan-associated toxicity.^8^ In addition, administration of drugs may precipitate shifts in the composition of the microbiota to favor the abundance of microbial taxa that have metabolizing capacity for those drugs. Pathological states, e.g., small intestinal bacterial overgrowth, introduce microbial metabolism into regions where it does not normally occur.^25^ Despite this, most studies treat the gut microbiome as spatially uniform, obscuring site-specific interactions critical for predictive translation. Emerging “smart” sampling approaches could help capture small intestine microbial activity,^26^ but their utility will depend on careful control of sampling site (luminal vs. mucosal, proximal vs. distal) and timing relative to drug exposure, to ensure measurements reflect biologically relevant activity rather than artefacts.

## Discussion

Drug-microbe interactions are robust and clinically relevant, yet their translation into predictive tools remains limited because current approaches measure static microbial features rather than context-dependent functional activity. Across mechanistic, cohort, and ecological studies, a consistent pattern emerges, where microbial contributions to drug efficacy and toxicity are not intrinsic properties of taxa or isolated enzymes, but emergent outcomes shaped by host physiology, spatial organization along the gastrointestinal tract, and community-level interactions. This mismatch between what is measured and what determines *in vivo* drug fate fundamentally constrains generalization across patients.

Functional readouts outperform taxonomic profiles precisely because they capture convergent microbial outputs that integrate ecological redundancy and host modulation. However, even functional outputs fail to generalize when measured without reference to the gut environmental conditions under which they are expressed. As a result, identical functional repertoires can produce divergent clinical outcomes across individuals, explaining why predictive markers often collapse when applied beyond the discovery cohort. Spatial organization further refines drug-microbiome interactions. Drug absorption and microbial metabolism are segregated along the gastrointestinal tract, such that site-specific exposure determines whether microbial transformation occurs at all. Alterations in transit, barrier function, or microbial localization can shift these spatial relationships, introducing or removing microbial drug metabolism in ways that profoundly affect efficacy and toxicity. Ignoring this spatial dimension effectively averages over biologically distinct niches, obscuring interactions that are critical for prediction. While functional readouts provide a more accurate picture of microbiome contributions to drug response, translating these measurements into clinical practice remains challenging. Microbiome sequencing is resource-intensive, both in cost and time, and data analysis is complex. In addition, consensus on what constitutes a “healthy” microbiome is still lacking. From a practical perspective, clinically actionable readouts are likely to focus on specific microbial functions, pathways or metabolites that reproducibly influence drug metabolism or response, rather than comprehensive taxonomic profiling.

Finally, drugs act as ecological forces that reshape microbial communities, and their adaptive capacity over time. Many non-antibiotic drugs often inhibit bacterial growth or alter microbial physiology, driving shifts in dominance and functional potential that can last years after treatment has ended.^27^ These ecosystem-level responses influence colonization resistance, drug-transforming activities and trigger stress responses that can alter antimicrobial resistance gene dynamics, introducing feedback that modify both immediate drug response and long-term outcomes. Ecological disruption has downstream clinical consequences. Loss of colonization resistance increases susceptibility to enteric pathogens.^28^^,^^29^ Chronic drug exposure enriches drug-transforming enzymes and antibiotic resistance genes.^1^^,^^30^^,^^31^ Because these ecological trajectories are host- and context-specific, they further limit the portability of predictive markers derived from static snapshots. Establishing causal links between microbiome function and drug response remains a major challenge. Mechanistic studies, including *in vitro* assays and animal models, have been invaluable for identifying potential microbial pathways involved in drug response and metabolism. However, these systems often do not capture the complexity of the human gut ecosystem, limiting their predictive value for clinical translation. Future approaches should focus on functional measurements *in situ* in humans, such as luminal or mucosal sampling, metabolomics, or stable isotope tracing, combined with longitudinal cohort data and computational models that integrate host physiology, spatial organization, and ecological interactions. Such strategies would allow identification of reproducible, context-dependent microbial functions that are more likely to serve as clinically actionable, predictive biomarkers. Together, these observations suggest that predictive pharmacomicrobiomics will require a shift from cataloging interactions to measuring where, when, and under which host conditions microbial functions are expressed.

## Conclusion

Drug–microbe interactions influence drug efficacy, toxicity, and long-term outcomes, yet translation fails because most studies treat microbial activity as static rather than context-dependent. Integrating functional outputs with spatially resolved microbiome data, host physiology and ecological dynamics offers a path toward patient-specific prediction. This approach will enable microbiome-informed personalized therapeutics and rational drug design, moving the field beyond documenting existence toward true clinical predictability.

## References

[1] Vich Vila A, Collij V, Sanna S, Sinha T, Imhann F, Bourgonje AR, Mujagic Z, Jonkers DMAE, Masclee AAM, Fu J, et al. Impact of commonly used drugs on the composition and metabolic function of the gut microbiota. Nat Commun. 2020;11(1):362. doi: 10.1038/s41467-019-14177-z. 31953381 PMC6969170

[2] Nagata N, Nishijima S, Miyoshi‑Akiyama T, Kojima Y, Kimura M, Aoki R, Ohsugi M, Ueki K, Miki K, Iwata E, et al. M. Population‑level metagenomics uncovers distinct effects of multiple medications on the human gut microbiome. Gastroenterology. 2022;163(4):1038–1052. 35788347 10.1053/j.gastro.2022.06.070

[3] Zimmermann M, Zimmermann-Kogadeeva M, Wegmann R, Goodman AL. Mapping drug–microbiome interactions in humans. Nature. 2019;570(7762):462–467. doi: 10.1038/s41586-019-1291-3. 31158845 PMC6597290

[4] Forslund SK, Chakaroun R, Zimmermann‑Kogadeeva M, Markó L, Aron‑Wisnewsky J, Nielsen T, Forslund SK, Zimmermann-Kogadeeva M, Aron-Wisnewsky J, Moitinho-Silva L, et al. Combinatorial, additive and dose‑dependent drug–microbiome associations. Nature. 2021;600:500–505. doi: 10.1038/s41586-021-04177-9. 34880489

[5] Kamath S, Chan NSL, Joyce P. GLP-1 agonists and the gut microbiome: a bidirectional relationship. Br J Clin Pharmacol. 2026. doi: 10.1002/bcp.70487. Online ahead of print. PMC13122291 41703894

[6] Maier L, Pruteanu M, Kuhn M, Zeller G, Telzerow A, Anderson EE, Brochado AR, Fernandez KC, Dose H, Mori H, et al. Extensive impact of non‑antibiotic drugs on human gut bacteria. Nature. 2018;555:623–628. doi: 10.1038/nature25979. 29555994 PMC6108420

[7] Michaelis L, Berg L, Maier L. Confounder or confederate? The interactions between drugs and the gut microbiome in psychiatric and neurological diseases. Biol Psychiatry. 2024;95(4):361–369. doi: 10.1016/j.biopsych.2023.06.004. 37331548

[8] Wallace BD, Wang H, Lane KT, Scott JE, Orans J, Koo JS, Venkatesh M, Jobin C, Yeh L, Mani S, et al. Alleviating cancer drug toxicity by inhibiting a bacterial enzyme. Science. 2010;330(6005):831–835. doi: 10.1126/science.1191175. 21051639 PMC3110694

[9] van Kessel SP, Frye AK, El GA, Castejon M, Keshavarzian A, van Dijk G, El Aidy S. Gut bacterial tyrosine decarboxylases restrict levels of levodopa in the treatment of parkinson’s disease. Nat Commun. 2019;10:310. 30659181 10.1038/s41467-019-08294-y PMC6338741

[10] Pereira FC, Ge X, Kristensen JF, Kirkegaard RH, Maritsch K, Szamosvári D, Imminger S, Seki D, Shazzad JB, Zhu Y, et al. The parkinson’s disease drug entacapone disrupts gut microbiome homoeostasis via iron sequestration. Nat Microbiol. 2024;9:3165–3183. doi: 10.1038/s41564-024-01853-0. 39572788 PMC11602724

[11] Klünemann M, Andrejev S, Blasche S, Mateus A, Phapale P, Devendran S, Vappiani J, Simon B, Scott TA, Kafkia E, et al. Bioaccumulation of therapeutic drugs by human gut bacteria. Nature. 2021;597:533–538. 34497420 10.1038/s41586-021-03891-8 PMC7614428

[12] Guantai LM, Bavinton CE, Shazzad JB, Mahajan S, Thompson S, Pereira FC. Taxonomic and mechanistic insights into gut microbiota bioaccumulation of entacapone using bioorthogonal drug labelling. Microbiome Res. Rep.. 2025;4:41. 41438002 10.20517/mrr.2025.73 PMC12719382

[13] Garcia-Santamarina S, Kuhn M, Devendran S, Maier L, Driessen M, Mateus A, Mastrorilli E, Brochado AR, Savitski MM, Patil KR, et al. Emergence of community behaviors in the gut microbiota upon drug treatment. Cell. 2024;187(22):6346–6357.e20. doi: 10.1016/j.cell.2024.08.037. 39321801

[14] Liang X, Bittinger K, Li X, Abernethy DR, Bushman FD, FitzGerald GA. Bidirectional interactions between indomethacin and the murine intestinal microbiota. eLife. 2015;4:e08973. doi: 10.7554/eLife.08973. 26701907 PMC4755745

[15] Subramaniam S, Elz A, Wignall A, Kamath S, Ariaee A, Hunter A, Newblack T, Wardill HR, Prestidge CA, Joyce P. Self-emulsifying drug delivery systems (SEDDS) disrupt the gut microbiota and trigger an intestinal inflammatory response in rats. Int J Pharm. 2023;648:123614. doi: 10.1016/j.ijpharm.2023.123614. 37979632

[16] Vinarov Z, Abdallah M, Agundez JA, Allegaert K, Basit AW, Braeckmans M, Ceulemans J, Corsetti M, Griffin BT, Grimm M, et al. Impact of gastrointestinal tract variability on oral drug absorption and pharmacokinetics: An UNGAP review. Eur J Pharm Sci. 2021;162 105812. 33753215 10.1016/j.ejps.2021.105812

[17] Peng Z, Cheng S, Kou Y, Wang Z, Jin R, Hu H, Zhang X, Gong J‑F, Li J, Lu M, et al. The gut microbiome is associated with clinical response to anti‑PD‑1/PD‑L1 immunotherapy in gastrointestinal cancer. Cancer Immunol. Res. 2020;8(10):1251–1261. doi: 10.1158/2326-6066.CIR-19-1014. 32855157

[18] Zhu X, Hu M, Huang X, Li L, Lin X, Shao X, Li J, Du X, Zhang X, Sun R, et al. Y. Interplay between gut microbial communities and metabolites modulates pan‑cancer immunotherapy responses. Cell Metab. 2025;37(4):806–823. doi: 10.1016/j.cmet.2024.12.013. 39909032

[19] Mimpen IL, Battaglia TW, Parra‑Martinez M, Toner‑Bartelds C, Zeverijn LJ, Geurts BS, Verkerk K, Hoes LR, van Renterghem AWJ, Noë M, et al. Microbial metabolic pathways guide response to immune checkpoint blockade therapy. Cancer Discov. 2026;16(1):95–113. 40996449 10.1158/2159-8290.CD-24-1669

[20] Artacho A, Isaac S, Nayak R, Flor‑Duro A, Alexander M, Koo I, Manasson J, Smith PB, Rosenthal P, Homsi Y, et al. The pretreatment gut microbiome is associated with lack of response to methotrexate in new‑onset rheumatoid arthritis. Arthritis Rheumatol. 2021;73(6):931–942. doi: 10.1002/art.41622. 33314800 PMC11293279

[21] Forslund K, Hildebrand F, Nielsen T, Falony G, Le Chatelier E, Sunagawa S, Prifti E, Vieira-Silva S, Gudmundsdottir V, Krogh Pedersen H, et al. Disentangling type 2 diabetes and metformin treatment signatures in the human gut microbiota. Nature. 2015;528:262–266. doi: 10.1038/nature15766. 26633628 PMC4681099

[22] Wu H, Esteve E, Tremaroli V, Khan MT, Caesar R, Mannerås-Holm L, Ståhlman M, Olsson LM, Serino M, Planas-Fèlix M, et al. Metformin alters the gut microbiome of individuals with treatment-naive type 2 diabetes, contributing to the therapeutic effects of the drug. Nat Med. 2017;23(7):850–858. doi: 10.1038/nm.4345. 28530702

[23] Cussotto S, Clarke G, Dinan TG, Cryan JF. Psychotropics and the microbiome: a chamber of secrets. Psychopharmacology (Berl). 2019;236(5):1411–1432. doi: 10.1007/s00213-019-5185-8. 30806744 PMC6598948

[24] Lima SF, Pires S, Rupert A, Oguntunmibi S, Jin WB, Marderstein A, Funez‑dePagnier G, Maldarelli G, Viladomiu M, Putzel G, et al. The gut microbiome regulates the clinical efficacy of sulfasalazine therapy for IBD‑associated spondyloarthritis. Cell Rep. Med. 2024;5(3):101431. 38378002 10.1016/j.xcrm.2024.101431 PMC10982976

[25] Bamba S, Imai T, Sasaki M, Ohno M, Yoshida S, Nishida A, Takahashi K, Inatomi O, Andoh A. Altered gut microbiota in patients with small intestinal bacterial overgrowth. J Gastroenterol Hepatol. 2023;38(1):61–69. 36180941 10.1111/jgh.16013

[26] Rehan M, Al-Bahadly I, Thomas DG, Young W, Cheng LK, Avci E. Smart capsules for sensing and sampling the gut: status, challenges and prospects. Gut. 2023;73(1):186–202. doi: 10.1136/gutjnl-2023-329614. 37734912 PMC10715516

[27] Aasmets O, Taba N, Krigul KL, Andreson R, Estonian Biobank Research Team, Org E. A hidden confounder for microbiome studies: medications used years before sample collection. mSystems. 2025;10(10), e00541-25. doi: 10.1128/msystems.00541-25. 40910778 PMC12542737

[28] Kumar A, Sun R, Habib B, Deng T, Bencivenga‑Barry NA, Palm NW, Ivanov II, Tamblyn R, Goodman AL. Identification of medication–microbiome interactions that affect gut infection. Nature. 2025;644(8076):506–515. 40670788 10.1038/s41586-025-09273-8 PMC13092348

[29] Grießhammer A, de la Cuesta‑Zuluaga J, Müller P, Gekeler C, Homolak J, Chang H, Schmitt K, Planker C, Schmidtchen V, Gallage S, et al. Non‑antibiotics disrupt colonization resistance against enteropathogens. Nature. 2025;644(8076):497–505. 40670795 10.1038/s41586-025-09217-2 PMC12350171

[30] Kim HB, Cho YJ, Choi SS. Metformin increases gut multidrug resistance genes in type 2 diabetes, potentially linked to escherichia coli. Sci Rep. 2024;14:21480. 39277620 10.1038/s41598-024-72467-z PMC11401871

[31] Shi D, Hao H, Wei Z, Yang D, Yin J, Li H, Chen Z, Yang Z, Chen T, Zhou S, et al. Combined exposure to non‑antibiotic pharmaceutics and antibiotics in the gut synergistically promote the development of multi‑drug resistance in escherichia coli. Gut Microbes. 2022;14(1):e2018901. doi: 10.1080/19490976.2021.2018901. PMC8757474 35014598

